# Supplementary material for: Are carbon emissions trading and green financial instruments synergistic? -Comprehensive quantitative research based on content analysis
Source: PLoS One. 2024 Mar 7;19(3):e0298601. doi: 10.1371/journal.pone.0298601 (PMC10919601; doi:10.1371/journal.pone.0298601)
Supplement: S1 Data — (ZIP) [file pone.0298601.s001.zip › Data/地级市Z_F工作报告.zip!/║╙─╧/╓▄┐┌╩╨/└┤╫╘╣1⁄2╓┌║┼í╛┬φ┐╦╩2╛▌═°í┐_2006.docx]

各位代表：

　　现在，我代表市人民政府，向大会作政府工作报告，请予审议，并请各位政协委员和其他列席人士提出意见。

　　一、2005年及“十五”工作回顾

　　2005年是我市经济增长又快又好的一年。一年来，市人民政府在省委、省政府和市委的正确领导下，认真贯彻落实科学发展观，以“项目推进年”为经济工作的切入点和总抓手，团结带领全市人民，扎扎实实打牢基础，千方百计加快发展，圆满完成了市一届人大六次会议确定的各项目标任务。

　　——经济总量取得新突破。初步核算，全市生产总值完成592.2亿元，增长11.4%。第一产业增加值205.3亿元，增长7%；第二产业增加值236.5亿元，增长15.1%；第三产业增加值150.4亿元，增长11.8%。

　　——工农业生产快速发展。规模以上工业增加值完成99亿元，实现利润18.2亿元，分别增长22%和113.2%。工业经济效益指数达164.2%；全市粮食总产552.3万吨，较上年增产11.2万吨，为国家粮食安全做出了新的贡献。

　　——投资保持高速增长。全社会固定资产投资完成222亿元，增长42.3%。其中，城镇固定资产投资完成139.3亿元，增长38%。

　　——财政金融实力明显增强。地方财政一般预算收入完成13.3亿元，增长31.8%。其中，市本级完成3.5亿元，增长33.5%。财政一般预算支出54.6亿元，增长30.1%。财政收支增幅均创历史新高；金融机构各项存款余额422.3亿元，较年初增加53亿元。其中，居民储蓄存款余额367.3亿元，较年初增加50.7亿元。金融机构各项贷款余额355.5亿元；保险业保费收入达到8.7亿元，向社会提供赔付金1.5亿元。

　　——内外贸易成果丰硕。社会消费品零售总额达到206.5亿元，增长14.2%；进出口总额2.3亿美元，增长9.7%；实际利用外商直接投资4865万美元，增长35%。

　　——就业再就业成效显著。全年新增城镇就业人员6.2万人；下岗失业人员实现再就业3.4万人，其中“4050”人员8900多人；城镇登记失业率3.69%。

　　——人民生活水平进一步提高。城镇居民人均可支配收入6356元，增长12.6%；农民人均纯收入2276元，增长11.1%。居民消费价格总水平上涨2.3 %。

　　2005年各项目标任务的胜利完成，标志着“十五”期间我市经济社会发展又跃上了一个新台阶。综合经济实力显著增强。国民经济实现了持续快速健康发展，生产总值年均增长8.3%。与2000年相比，经济总量增长50%，地方财政一般预算收入和支出均实现翻番。结构调整迈出实质性步伐。二、三产业比重达到65.3%，比“九五”末提高4.5个百分点。农副产品精深加工基地初具规模。五年累计转移农村人口87万人。城镇化率达到19%，提高6个百分点。项目建设成效显著。五年完成固定资产投资689亿元，年均增长23.1%，其中，城镇基础设施投资211亿元。去年，3000万元以上在建项目138个，其中,亿元以上在建项目53个。公路通车里程五年新增6100公里，发电装机容量新增27万千瓦。对外开放取得新成效。五年累计进出口总额9.8亿美元，是“九五”期间的2.8倍，其中，出口3.4亿美元；实际利用外商直接投资1.2亿美元。人民群众得到较多实惠。居民消费价格总水平基本稳定，城镇居民人均可支配收入、农民人均纯收入年均增长11.8%和3.5%，城乡居民人均居住面积分别达到29.3和21.7平方米。社会事业全面进步。科技进步对经济增长的贡献率进一步提高；普通高校在校生达到1.4万人，比“九五”末增长2.9倍多；医院病床数12435张，比“九五”末增长4.7 %；人口自然增长率低于全省平均水平。经过五年的努力，我市经济社会已经步入一个新的发展阶段。

　　回顾去年及“十五”的工作，主要做法和体会是：

　　(一)坚定不移地以科学发展观统领经济社会发展全局，着力推进“三化”进程

　　积极探索传统农区的小康建设道路，坚持不懈地加快工业化、城镇化，推进农业现代化。立足于我市农副产品资源和劳动力资源丰富的优势，全力打造农副产品精深加工基地，不断推进产业结构优化升级。五年共统筹使用工业结构调整和高新技术产业化贴息资金6500万元，支持了一批对产业结构升级具有示范带动作用的工业项目。农副产品加工业占全市工业的比重达到60%。加快培育企业集团。12家企业分别被评为全国和全省农业产业化重点龙头企业。2家企业被确定为第一批全国农产品加工业示范企业。益海粮油公司跻身全国外资企业百强。7家企业入围全省百户重点工业企业，2家被评为全省高成长性企业，7家被评为全省优秀民营企业。去年全市规模以上工业企业实现利税总额29.4亿元，产品销售收入299亿元，分别比2000年增长1.6倍和1.2倍 ；全市销售收入超10亿元的工业企业达到5家，莲花集团销售收入突破38亿元。以撤地设市为契机，积极构建辐射带动全市的三级城镇体系，推动城乡统筹协调发展。五年城镇建成区面积新增112平方公里，城镇人口新增87万人。累计完成城市基础设施建设投资69亿元，新增城市道路525公里。随着城市总体规划和控制性详规的实施，周口师院新校区，市中心血站大楼等一批社会事业项目建成投用。文昌大道、中原大道、沙南污水处理厂等一批项目建成或开工，城市基础设施和城市面貌明显改善，中心城市框架逐步拉大，功能日益完善，发展活力不断增强。一个产业发展、环境优美、人与自然和谐相处的区域性中心城市初具规模。4个县(市区)通达高速，6个县城和86%的乡镇实现了二级公路联系，城镇化进程明显加快，辐射和带动经济发展的能力增强。认真落实支农政策，加快农业结构调整，稳步提高农业综合生产能力。全面落实“一免三补”政策，仅去年各项支农支出就达3.5亿元，比上年增长58.9%。4个县被批准为国家大型商品粮基地，5个县被列入全省农业综合开发重点县，9个县市被列入国家优质粮食产业工程县，4个县跻身全国粮食生产百强县，7个县分别被列入全国棉花和全国油料生产百强县。已建成97个省级优质无公害农产品基地，扶沟县被确定为国家级无公害蔬菜生产基地县，2个农场被确定为国家级无公害农产品示范农场。林业生态状况进一步改善，全市整体实现平原绿化高级达标。畜牧业发展步伐加快，畜牧业产值占农业总产值的比重达36%，鹿邑和太康分别被确定为全国畜产品加工业示范基地和全省无公害畜产品生产示范基地。农产品加工转化能力不断增强，粮食、肉类、乳品加工能力分别达到400万吨、30万吨和5万吨。大力发展劳务经济，形成了“海燕技工”、“项城防水”、“西华的哥”、“商水摘棉工”等一批在全国有影响的劳务品牌。去年外出务工人员达到226万人次，劳务收入已成为农民增收的重要渠道。同时，着力加快旅游、文化、通信、现代物流等新兴服务业发展。去年接待海内外游客突破450万人次，旅游总收入达到1.86亿元；文化产业完成增加值4573万元，增长4.68%；邮政业务总量完成1.76亿元，比上年增长9.33 %；移动和固定电话用户分别达到142万户和106.9万户，宽带用户达到4.1万户,通讯能力和质量全面提高。

　　(二)坚定不移地以项目建设为总抓手，着力为经济社会发展打牢基础

　　连续二年开展“项目建设年”和“项目推进年”活动，着力加快国家鼓励发展的农业、水利、交通等基础产业和基础设施建设。引导金融机构加大信贷投放，积极鼓励、引导社会资金投入，建成和开工了一大批事关经济社会发展全局的重大建设项目，培育了一批新的经济增长点，为经济社会长远发展提供了有力支撑。去年，争取226个国债资金、中央预算内资金和省专项资金项目，总投资42.6亿元，其中，国家和省各类资金10.3亿元。民间投资比重已达53 %，比2000年提高21个百分点；工业投资58亿元，是2000年的4.2倍。益海、隆达、天瑞、五得利、华林等一批骨干企业投产运营；总投资7100万元的煤及煤层气勘探进展顺利；漯周界高速公路周口段建成通车，大广、周商、许亳3条高速周口段相继开工；漯阜铁路周口段改造完成，开行了周口直达北京的旅客列车；沙颍河复航工程竣工，实现季节性通航。我市公路、铁路、水路三位一体的大交通格局基本形成。沙河、涡河治理改造先后开工，一批农田水利工程相继投用，防洪除涝能力明显提高，去年再夺全省“红旗渠精神杯”大奖。

　　(三)坚定不移地推进改革开放，着力增强经济社会发展活力

　　国有企业改革进一步深化。以产权制度改革为核心，积极引进战略投资者，大力发展混合所有制经济，86%的国有工业企业实现股权多元化，组建股份制公司147户，累计盘活国有资产49.8亿元，吸纳引进社会资本及改制后的再投入5.2亿元。莲花味精集团通过改制实现了产销两旺，周口鑫达纺织有限公司目前具备10万锭生产能力，3000多名职工重新上岗。国有企业下岗职工全部出再就业服务中心。非公有制经济快速发展。制定了促进非公有制经济加快发展的政策措施，不断优化发展环境，非公有制经济占全市经济总量的比重己达52%。农村税费改革取得阶段性成果。全面取消了乡统筹、农村教育集资等专门面向农民征收的行政事业性收费和政府性基金，取消了除烟叶以外的农业特产税，全部免征农业税，全市农民累计减负增收达13.8亿元；实施对种粮农民直接补贴、良种补贴和大型农机具购置补贴，调动了农民种粮积极性。粮食流通体制改革圆满完成。全面放开粮食购销市场和价格，累计分流安置富余人员24917人，125家企业退出国有粮食购销企业序列，新组建国有独资企业12家、国有控股企业131家，粮食购销市场主体多元化格局初步形成。农村信用社改革进展顺利。10家县级联社全部获得专项央行票据认购权，增资扩股8.2亿元。管理水平明显提高，经济效益整体好转。机构改革和行政审批制度改革成效明显。通过新一轮市县机构改革，政府机构、人员编制分别精简11.1%和16.5%；乡镇机构改革顺利完成，减少乡镇行政事业机构305个，清退临时人员1542人，分流超编人员22403人。清理规范行政审批事项，取消行政许可项目1162项、收费项目315项。在项城市、鹿邑县成为省扩权市县之后，又扩大了鹿邑县玄武镇、沈丘县付井镇等9个重点乡镇的经济和部分社会管理权限，为县域经济加快发展提供了良好的体制保障。与此同时，交通、建设、水利、科技、教育、文化、卫生等领域的改革都迈出了新步伐。

　　适应加入世贸组织后的新形势，积极实施开放带动主战略，加快发展开放型经济。五年来已与103个国家和地区建立了经贸关系，48家外资企业落户周口，新加坡丰益集团、北京三吉利、平顶山天瑞、河北五得利、湖南唐人神等一批国内外大企业投资我市。近三年引进市外资金287亿元，去年超过126亿元。周口海关正式开关运行，市经济开发区顺利通过国家设立核准，为我市对外开放打造了新的平台。派100多名干部到省直机关、省管大型企业、沿海地区挂职锻炼、驻地招商，促进了招商引资和对外开放。

　　(四)坚定不移地坚持以人为本、执政为民，着力解决好涉及群众利益的突出问题

　　认真做好就业和社会保障工作，五年城镇新增就业人员25.8万人，下岗失业人员再就业11.5万人，其中“4050”人员2.5万人。巩固“两个确保”，规范和完善城市“低保”，企业职工基本养老保险实行市级统筹，国有企业下岗职工基本生活费、失业人员失业保险金、企业离退休人员养老金做到了按时足额发放，10.9万城市低保对象基本生活得到保障。完善农村五保供养制度，建立了农村五保供养新机制。全面实施农村特困户救助，17.7万特困群众享受救济。进一步加大农村公路建设投入，仅2003年—2005年就筹措资金10.5亿元，新建和改造农村公路4336公里，去年新增通油路行政村682个。投资9539万元，解决了重污染地区352个村、50多万人的饮水安全问题；投资3200万元，建成第三水厂、周口傍河水源地及其管网配套设施，结束了周口市民世世代代吃沙河水的历史。五年来，累计投入危改资金2.3亿元，改造学校1862所，消除农村中小学D级危房41.2万平方米，提前一年超额完成省定改造任务。去年投入资金1912万元，对74所中心卫生院进行改造；在太康县开展了新型农村合作医疗试点，参合农民达83.8万人。认真落实农村义务教育“两免一补”政策，去年投入1.8亿元资助农村贫困家庭中小学生94万人。加大扶贫工作力度，五年来共有66.8万农村贫困人口脱贫。扎实做好救灾工作，2003年以来，帮助受灾群众重建住房2.4万余间。全面完成城市和农村电网改造，实现了城乡居民生活用电同价。去年新建沼气池20.2万个，建成生态文明村116个，促进了农村经济发展和村容村貌改善，提高了农民生活质量。加大清理建筑领域拖欠工程款和农民工工资工作力度，累计清理拖欠工程款和农民工工资分别为4.4亿元和6553万元，维护了进城务工人员合法权益。

　　(五)坚定不移地坚持统筹协调发展，着力促进社会全面进步

　　强化政府社会管理和公共服务职能，努力促进社会全面进步。社会事业加快发展。坚持科技进步与创新，大力发展高新技术产业，实施科技富民、粮食丰产、制造业信息化等科技工程，获得省级以上科技成果奖励32项、国家授权专利1000项；27家企业分别被命名为国家级和省级高新技术企业，5家企业获省制造业信息化示范企业称号，5家企业跻身全省民营科技企业50强行列；五年来开发农作物新品种40多个，科技对经济发展的支撑能力进一步增强。巩固提高“两基”成果，提升高校办学水平，大力推进职业教育和民办教育，各级各类教育全面协调发展，编制内农村中小学教师工资按时足额发放。周口师院专升本，周口职业技术学院实行了5校区实质性合并。夺取抗击“非典”胜利之后，先后开工建设了市传染病医院、8个县级传染病区、市紧急救援中心、市中心医院病房大楼、市中医院门诊病房综合楼、疾病预防控制中心大楼等公共卫生基础设施，疾病预防控制和医疗救治体系进一步完善。加快文化产业和文化事业发展，成功举办了首届中华姓氏文化节、“公祭人文始祖伏羲大典”和以“自然·和谐·发展”为主题的弘扬老子文化国际研讨会，我市被命名为中国“杂技之乡”；实施文化精品工程，《都市霓虹》、《远山春雨》等一批文艺精品剧目荣获省级大奖。广泛开展全民健身活动，连续五年被评为全国全民健身先进市。竞技体育水平不断提高，五年来共在省级以上比赛中获得金牌103枚。审计、监察监督进一步强化。统计、物价、外事、侨务、气象、人防、史志等各项社会事业取得全面发展。基本国策得到落实。深入开展计划生育清查处理和综合整治工作，扭转了计划生育被动局面。实施农村计划生育家庭奖励扶助政策，去年6726人享受到奖励扶助。在严格土地管理的同时，大力开展“空心村”、砖瓦窑场、工矿废弃地“三项整治”，去年新增耕地3776.7公顷，基本保障了建设用地需要。不断加大对重点排污企业的监控和治理力度，重点查处“十五小土”企业，高度重视生态环境保护建设工作。五年来，全市共关闭、取缔不符合国家产业政策污染严重的企业126家，整体环境质量明显改善。安全生产形势逐步好转。不断强化安全生产工作，事故起数和死亡人数呈逐年下降趋势。精神文明和民主法制建设得到加强。广泛开展“道德规范进万家、诚实守信万人行”和“双百”评选活动，深入开展文明卫生城市创建工作，积极开展向陈新庄、洪战辉等先进人物学习活动，大力推进未成年人思想道德建设及伦理、心理、生理教育，城乡文明程度不断提高。继续推进基层民主政治建设，顺利完成第四、第五届村委会换届选举。政务公开、厂务公开和村务公开不断扩大。加强法制宣传教育，完成了“四五”普法任务。自觉接受人大法律监督、工作监督和政协民主监督，认真办理人大代表建议和政协委员提案。加强政府廉政建设，反腐败斗争取得新成效。深入开展双拥工作，巩固军政军民团结，项城企业军转干部稳定工作经验得到中央肯定，在全省、全国推广学习。去年，我市被命名为省级双拥模范城。加强社会治安防控体系建设，严厉打击各类违法犯罪活动，打击遏制“两抢一盗”专项斗争取得显著成效，荣获2005年度全省社会治安综合治理工作先进市称号。深入开展民族团结进步教育活动，引导宗教与社会主义社会相适应，民族宗教工作得到进一步加强。依法做好信访工作，建立健全应急机制，社会大局保持稳定。

　　各位代表，过去的五年，是我市经济取得突破性进展，经济质量明显提高，人民群众得到实惠最多的五年；是统筹城乡发展力度最大，城乡面貌改变最快，城市承载能力不断提高的五年；是社会事业全面发展，精神文明和民主法制建设不断取得进步的五年。五年来的主要体会是：实现经济社会快速协调发展，必须坚持以科学发展观统领经济社会发展全局，切实做到“四个融会贯通”，即：把坚定不移地坚持科学发展观融会贯通到我市经济社会发展的各个方面，转变观念，与时俱进，实现经济社会更快更好地发展；把坚定不移地坚持宏观调控融会贯通到我市改革开放和现代化建设的全过程，顺势而为，乘势而上，在调控中加快发展；把坚定不移地坚持改革创新融会贯通到全市经济结构战略性调整的始终，突出重点，稳步推进，在优化结构中加快发展；把坚定不移地坚持以人为本融会贯通到全市各项重大决策和重要工作部署中去，更加关注人民生活，在构建和谐社会中加快发展。必须坚持把招商引资和项目建设作为加快经济社会发展的重要推动力量，不断改善经济发展环境和条件，促进投资主体多元化，增强经济发展的活力和后劲。必须坚持一切从实际出发，理清发展思路，发挥自身优势，牢牢把握发展的主动权。必须坚持大力弘扬求真务实之风，不断强化工作责任，狠抓工作落实，确保各项工作取得实效。必须充分调动方方面面的积极性，凝聚广大干部群众的智慧和力量，团结一致，齐心协力，奋力拼搏，努力形成百舸争流、竞相发展的良好局面。

　　各位代表，五年来之所以取得这些成绩，是省委、省政府和市委正确领导的结果，是人大、政协积极支持的结果，也是各县市区、各部门和全市广大干部群众共同奋斗的结果。在此，我代表市人民政府，向工作在全市各条战线上的广大工人、农民、知识分子、干部、人民解放军指战员、武警官兵以及各界人士致以崇高的敬意！向驻周单位及所有关心、支持我市现代化建设的同志们、朋友们，表示衷心的感谢！

　　各位代表，五年的发展成就令人鼓舞，但我们必须清醒地看到，我市经济社会还面临许多亟待解决的矛盾和问题。主要是经济结构不合理；工业规模小、总量少，发展后劲不足；农业综合生产能力不高，农民增收任务艰巨；城市化率低，辐射带动能力不强；生产和建设资金严重不足；就业和社会保障压力较大，维护社会和谐稳定的工作任务繁重；投资环境需要进一步改善等。对此，我们将高度重视，在今后的工作中，通过加快发展和深化改革着力加以解决。

　　二、“十一五”发展的基本思路和工作重点

　　“十一五”是我市承前启后、实现跨越式发展的关键时期。根据党的十六届五中全会精神和中共周口市委关于“十一五”规划建议，我市经济社会发展的基本思路是：坚持以邓小平理论和“三个代表”重要思想为指导，坚持以科学发展观统领经济社会发展全局，坚持以经济结构战略性调整为主线，坚持加快发展和打牢基础两个根本点，坚持把不断提高人民生活水平作为一切工作的出发点和落脚点，坚持立足“三农”抓“三农”与跳出“三农”抓“三农”相结合,大力实施工业兴市、开放带动、中心城市和城镇带动、科教兴市和人才强市“四大战略”，努力打造“一中心、两带、三基地”，着力建设粮食生产核心区和区域性交通枢纽。进一步加快工业化、城镇化，推进农业现代化，大力发展文化产业和人力资源产业，转变经济增长方式，完善体制机制，构建和谐社会，全面增强综合实力、发展活力和区域竞争力，开创我市社会主义经济建设、政治建设、文化建设和社会建设的新局面。

　　“十一五”经济社会发展的主要奋斗目标是：

　　——经济持续快速协调健康发展，综合实力再上新台阶。生产总值年均增长12%左右；提前2到3年实现人均生产总值比2000年翻一番；经济结构进一步优化，二、三产业的比重显著提高；城镇化水平达到35%左右。

　　——体制机制不断创新，开放型经济建设取得新突破。坚持市场化取向改革，社会主义市场经济体制更加完善。非公有制经济比重达到68%左右；五年累计利用外资3亿美元以上，2010年外贸进出口总额4亿美元以上。

　　——生态环境得到改善，资源节约型和环境友好型社会建设取得明显进展。单位生产总值能耗比“十五”末降低20%左右，主要污染物排放量削减5%，林木覆盖率达到25%以上，可持续发展能力不断增强。

　　——社会全面进步，和谐社会建设取得显著成效。科技进步贡献率达到45%左右，教育水平进一步提高，文化强市建设取得突破性进展，各项社会事业蓬勃发展。城镇居民人均可支配收入年均增长8%，农民人均纯收入年均增长6%。社会就业比较充分，社会保障体系比较健全，城乡居民生活质量和水平进一步提高。民主法制和精神文明建设进一步加强，公民的思想道德素质、科学文化素质和健康素质明显提高。

　　在新的发展时期，我们必须花更大的精力逐步把经济社会发展转入科学发展的轨道。要在以下四个方面做出更大努力：

　　(一)加快经济结构调整和增长方式转变

　　进一步转变发展观念，创新发展模式，提高发展质量。大力实施“工业兴市”战略，做强做大工业经济。发挥农副产品资源丰富的优势，培育八大支柱产业，坚持走新型工业化道路，努力整合各种优势资源，扩大工业经济总量，提升工业经济质量，加快结构调整，完善工业布局，改善工业经济发展环境，实现全市经济历史性跨越。坚持以投资拉动经济发展为主，扩大固定资产投资规模。继续推动基础设施建设和基础产业发展，建成一批关系国民经济和社会发展全局的水利、交通、能源、信息等重大项目，继续加强城建、环保、社会事业等薄弱环节建设，实施一批结构调整和产业升级项目，培育一批产业集群和产业基地，进一步增强经济社会发展后劲。积极发展第三产业，推动三次产业协调发展。在继续稳定提高农业综合生产能力、推进工业结构优化升级的同时，加快发展旅游、物流、文化、金融、保险、信息等现代服务业，改造提升商贸、交通运输、房地产等传统服务业，争取服务业增加值和从业人员所占比重有较大提升。切实增强自主创新能力，促进经济发展由农产品资源主导型向创新主导型转变。不断加大财政投入，加强技术创新体系建设，强化企业自主创新主体地位，大力发展高新技术产业，改造提升传统产业，增强科技对经济发展的支撑能力。扎实推进资源节约和综合利用，促进经济发展由粗放型向集约型转变。积极发展循环经济，大力推进节能降耗，切实提高资源利用效率。加强生态环境保护，坚持节约发展、清洁发展、安全发展和可持续发展，努力建设资源节约型、环境友好型社会。

　　(二)统筹城乡协调发展

　　坚持工业反哺农业、城市支持农村和多予少取放活的方针，统筹城乡发展，逐步建立以工促农、以城带乡的长效机制，突出抓好“两带三点”建设，即：周项淮经济隆起带、鹿郸沈沿边开放经济带及周口与许昌、开封、商丘三市公路交界点的新农村建设试点工作。建好太扶西优质农产品供应和精深加工示范区，构建城乡区域协调发展的大格局。实施中心城市和城镇带动战略，加快城镇化进程。坚持中心城市、县城、小城镇“三头并举”的方针，强化中心城市地位，加快新区、经济开发区、老城区建设，实现周商一体化，推进周淮一体化,改变中心城市一市一区格局，提升城市综合经济实力，增强辐射带动能力。大力发展县域经济，促进有条件的县市发展成为具有较强经济活力的中等城市。按照生产发展、生活宽裕、乡风文明、村容整洁、管理民主的要求，加快社会主义新农村建设。以发展农村经济为中心，进一步发展和解放农村生产力，促进农业稳定发展、农民持续增收。调整国民收入分配格局，不断加大对农业农村发展的投入，扩大公共财政覆盖农村的范围，促进基础设施向农村延伸、公共服务向农村拓展、社会保障向农村覆盖。搞好村庄规划和人居环境治理，逐步改善村容村貌。

　　(三)实施开放带动主战略

　　充分认识我市仍处于由农业经济主导型向工业经济主导型加速转变、农村经济主导型向城市经济主导型加速转变、封闭内向型经济向开放带动外向型经济加速转变时期的现实，认真解决存量有限、增量不足的问题，把招商引资和项目建设作为“总抓手”，发展开放型经济，不断提高对外开放水平。千方百计招商引资。把招商引资作为实现跨越式发展的长期战略举措，创新招商方式，打造招商平台，构筑招商网络。建立各级专业招商队伍，坚持和完善干部外派锻炼、招商引资的长效机制，增强招商引资的针对性，提高招商实效。提升对外开放水平。抓住国内外产业快速转移、资本和技术加速流动的难得机遇，发挥比较优势，利用好“两个市场”、“两种资源”，培育发展一批具有自主知识产权的国际、国内知名品牌产品，造就一批熟悉外经外贸工作的专业人才。继续扩大与发达地区的合作，加强与中西部省份的交流，进一步开展对外经贸合作和交往，努力扩大外贸进出口，不断拓展新的发展空间。

　　(四)推进和谐社会建设

　　更加关注民生，进一步解决经济社会发展不协调问题。千方百计扩大就业。积极发展就业容量大的服务业、劳动密集型企业和中小企业，形成经济增长与扩大就业良性互动的发展模式；建立城乡统一的劳动力市场，建立健全城乡就业公共服务网络；加强失业调控，把失业规模控制在社会可承受的范围之内。建立和完善与经济发展相适应的社会保障体系。进一步完善城镇基本养老、失业、医疗保险和城市居民最低生活保障制度。基本建立农村社会养老保险制度和最低生活保障制度；逐步扩大新型农村合作医疗试点，到2010年基本覆盖农村居民；健全覆盖城乡的社会救助体系。努力提高城乡居民收入水平。不断增加城镇低收入者和广大农民的收入，扩大中等收入者比重，努力缓解部分社会成员收入分配差距扩大的趋势。加快推进收入分配制度改革，鼓励各种生产要素按贡献参与分配。加快发展社会事业。加大公共服务投入，健全公共服务体系。新增教育、文化、卫生等事业经费主要用于农村，大力发展各项社会事业，促进人的全面发展和社会全面进步。

　　《周口市国民经济和社会发展第十一个五年规划纲要(草案)》及说明已印发各位代表，请予审议。

　　三、2006年工作的主要目标和任务

　　2006年是“十一五”规划开局之年。做好今年的工作，对于保持经济社会发展良好势头，为“十一五”开好头、起好步具有重要意义。今年我市经济社会发展的主要预期目标是：生产总值增长12%；全社会固定资产投资增长22%；社会消费品零售总额增长12%；外贸出口增长16%，实际利用外商直接投资增长25%；地方财政一般预算收入增长12%；城镇居民人均可支配收入增长8%，农民人均纯收入增长6%；居民消费价格总水平涨幅控制在3%以内；单位生产总值能耗降低4%左右；人口自然增长率控制在6‰以下；城镇新增就业5万人；城镇登记失业率低于4.5%。

　　围绕上述目标，重点抓好8个方面的工作：

　　(一)扎实推进社会主义新农村建设

　　贯彻工业反哺农业、城市支持农村的方针，落实各项支农惠农政策，以生态文明村建设为载体，以沼气建设为切入点，使新农村建设有个良好开端。

　　发展现代农业。围绕提高农业综合生产能力和农产品竞争力，搞好结构调整、科技进步和设施建设，发展优质、高产、高效、生态、安全、品牌农业。全力抓好9个县优质粮食产业工程。投资8000万元，做好商水、淮阳、郸城、鹿邑、太康5个全省农业综合开发重点县的开发工作。改造中低产田21.5万亩。投资6750万元，重点抓好郸城、淮阳、沈丘、鹿邑4个县国家大型商品粮基地续建项目，稳定和提高粮食综合生产能力。加快推进优势农产品的区域化布局、规模化生产和产业化经营。大力发展良种业，把我市建成黄淮流域以小麦、棉花为主的重要良种繁育基地。扩大特色经济作物种植面积，形成一县一品、一乡一品或一村一品,靠规模和特色创效益。因地制宜发展林业生产，扩大名特优经济林面积，提高林业综合效益。支持沈丘肉羊、奶牛基地建设，建立健全动物疫病防治体系，实现畜牧养殖业标准化生产，养殖小区发展到250个，规模养殖场发展到350个。坚持以工业理念发展农业，积极扶持财鑫、志元等农业产业化龙头企业，抓好现有的2个国家级、10个省级和88个市级农业产业化龙头企业的监测运行，争取有更多的企业纳入国家和省重点扶持的盘子。加强农产品质量检测和认证体系建设，完善农业技术推广和信息服务体系，推进农业标准化、产业化。引导和扶持农村合作经济组织、农产品行业协会发展，提高农民进入市场的组织化程度。加强农村现代流通体系建设，鼓励连锁超市由城市向农村延伸。

　　加强农村基础设施建设。抓住国家和省加大对农村发展、对新农村建设支持的重要机遇，积极争取各项支农资金，进一步改善农民生产生活条件，突出抓好沼气建设、饮水安全、村村通油路、农村信息化等重点工作。新增沼气用户20万户，结合养殖业和沼气发展，建设一批秸杆综合利用小区。解决重污染地区15万农民安全饮水问题。改造和新建农村公路1200公里，确保900个行政村通油路(水泥路)。扩大农村广播电视和信息网络覆盖面，建设党员远程教育工程试点，建成覆盖全市的农村信息网。投入3.5亿元，继续实施沙颍河、涡河综合治理续建工程。搞好农田水利、节水农业、林网建设等农村“六小”工程项目建设。

　　推进农村综合改革。继续抓好乡镇机构、农村义务教育和县乡财政体制等综合配套改革，积极探索化解乡村债务的办法和机制，巩固农村税费改革成果。继续对种粮农民实施直接补贴、良种补贴，稳定粮食价格，控制农资价格，防止农民负担反弹。建立财政保障机制，落实好村级经费和村干部报酬。扩大农业投入来源，继续对农户购买大型农机具实行补贴，动员和组织农民对直接受益的农田水利设施投工投劳，鼓励和引导各种社会资本投向农业。深化土地征用制度改革，健全对被征地农民的合理补偿和安置机制，切实保障农民的权益。

　　建设社会主义新农村是一项长期而艰巨的任务。要坚持以发展农村经济为中心，以增加农民收入为出发点，以解决农民最关心、最直接的问题为切入点，以改革开放为动力，充分调动各方面的积极性；要坚持从实际出发，因地制宜，分类指导，搞好规划；要坚持以农民为主体，充分尊重农民意愿，不搞形式主义和强迫命令，防止一哄而起；要发扬自力更生、艰苦奋斗精神，求真务实，真抓实干。

　　(二)积极开展“招商引资和优化环境年暨金融生态环境建设年”活动

　　进一步加大招商引资力度。积极承接国际和东部沿海发达地区产业转移，千方百计引进跨国公司、国内知名企业特别是世界500强企业在我市投资。抓好经济开发区和县市区工业园区建设，搭建招商引资平台。不断改进招商方式，提高招商引资实效。突出招商重点区域，瞄准长三角、珠三角、环渤海经济圈等发达地区，实施驻地招商，强化跟进措施，提高招商成功率。进一步充实优化专业招商队伍，加强业务培训，落实招商任务。坚持行政推动和市场化招商相结合，强化招商引资目标责任制和考核激励机制。全年力争实际利用外资5000万美元，引进内资150亿元。

　　大力发展外向型经济。积极拓展对外经济发展空间，努力扩大对外贸易和经济技术合作，调整优化出口商品结构，提高出口产品的科技含量和附加值。培植出口创汇骨干企业。大力发展加工贸易。落实出口退税政策，巩固扩大传统大宗农产品出口。积极引进国外先进技术和管理经验，扩大对外工程承包与劳务合作，参与国际市场竞争。

　　改善和优化发展环境。在继续加强硬环境建设的同时，重点抓好软环境建设。从影响投资和发展的突出问题入手，简政放权，减少审批环节，进一步完善“一个窗口对外”、“一站式”办公服务，落实首问负责制、承诺制和限时办结制等服务制度，为各类投资者提供便捷、周到、高效的服务。严肃查处“四乱”案件以及检查、管理中的“乱作为”、“不作为”、“慢作为”等严重影响经济发展环境的问题。坚持德治与法治并重，坚决打击无理取闹、强装强卸、欺行霸市、敲诈勒索的行为，努力营造公平规范的法制环境、优质高效的服务环境、诚信文明的人文环境。

　　营造安全共赢的金融生态环境。以集中整治逃废金融债务为突破口，以建立和完善社会诚信体系为重点，在着力抓好重点行业、重点企业信用建设的基础上，建立社会信用信息共享制度和监管机制，全面推动金融生态环境改善，逐步实现政府与银行、政府与企业、银行与企业之间多层次相互支持、良性互动的金融生态环境。

　　(三)大力发展二、三产业

　　强力实施工业兴市战略，做强食品加工、纺织服装、医药制造、皮革皮毛、板材家具、电力能源、化工建材、机械制造八大支柱产业，积极推动二、三产业互动，不断提高工业经济的带动力和竞争力。

　　加快工业园区建设。引导产业向园区聚集，工业向园区集中。加快市经济开发区基础设施建设，尽快形成食品、医药、能源、建材及高新技术专业园区，拉大发展框架，打造全市核心工业区。加快中小企业创业园区建设，发挥中小企业创业园区的“孵化器”作用，提高中小企业创业成功率。发展特色工业园区，扎实推进扶沟纺织工业园区建设。结合土地“三项整治”，抓住撤销城关镇设立办事处的有利时机，建成一批设施配套、功能齐全、企业集聚、带动发展的县市区工业园区，走企业集约发展道路。

　　突出抓好工业项目。今年我市投资3000万元以上重点工业项目39个，计划投资27.3亿元。按照前期项目抓论证、抓报批，意向项目抓签约、抓落地，在建项目抓服务、抓进度，建成项目抓运行、抓达产的要求，分类指导，加快五得利年产50万吨小麦深加工、中方10万纱锭生产线、万园肉牛年养殖1万头及年分割10万头等一批成长性较好的续建工业项目的建设步伐，建成益海年产30万吨面粉加工、金丹年产1.5万吨医药乳酸、唐人神年产30万吨饲料、龙源年产10万吨箱板纸扩建等12个工业项目，下大力气抓好科信电缆、桂邦食品、秸秆发电等新上工业项目，积极扶持施达肉牛育肥及加工、财鑫粉煤汽化、裕隆发电有限公司年产60万立方米灰砂砖等一批具有较高资源利用率、较低污染排放率的生产性项目。进一步加大投入，加快煤炭勘探进度。对去年已建成投产的天瑞水泥粉磨站等工业项目，加大协调服务力度，帮助企业开足马力，组织生产，使其尽快成为我市新的经济增长点。

　　加快发展文化和人力资源两大产业。以伏羲文化、老子文化、姓氏文化和中国杂技之乡的资源优势为依托，大力发展以文化资源开发、文化产品经营、文化设施利用为主的文化产业。利用我市厚重的历史文化资源和民间文化资源，办好第二届中华姓氏文化节、国际老子文化节和淮阳人祖庙会，使“两节一会”成为在全国有一定影响的知名节会。以“周、淮、鹿”三点一线旅游带为主轴，加大对太昊陵、太清宫、明道宫、平粮台遗址、女娲城、大程书院、吉鸿昌纪念馆、袁世凯故居等人文景观和历史文化遗存的保护修缮力度，综合开发人文旅游精品，带动旅游产业发展。全面整合我市文化资源，挖掘传统文化内涵，打造文化产业优势。加大人力资源投入，创新人才工作机制，为加快我市发展提供强大的人才保障与智力支持。按照培养有文化、懂技术、会经营的新型农民要求，充分发挥周口职业技术学院、海燕职专等职业教育和培训学校的桥梁作用，实施好“阳光工程”，切实把农村劳动力培训作为加大农村劳动力转移就业、增加农民收入的一项基础性工作来抓，变人口大市为人力资源大市，变劳力输出大市为技工输出大市。全年对26.5万农村劳动力分别进行职业、基本技能以及实用技术培训，转移农村富余劳动力240万人次。

　　积极发展服务业。改造提升交通运输、仓储、邮电通信、批发零售、餐饮等传统服务业。积极发展金融、保险、会计、审计、法律咨询、社区服务等现代服务业和新兴服务业。完善沙河周口港配套设施，使其尽快发挥效益。开工建设高速客运站。建成中都财富商城、项城市建材大市场，加快麒麟荷花商贸广场、铁路建材城、豫东南医药物流中心建设进度，搞活中原国际商贸城、时代广场，继续对荷花市场及其它专业市场进行整顿、规范和完善。实施“万村千乡”市场工程，在去年项城市试点的基础上，今年再增加5个县，建成1200个农家店，方便农民购物，促进农村商品流通。多方筹措资金，规划建设一批县域综合农业生产资料市场、乡镇生产资料配送中心和网点。充分挖掘我市一千多万人口的巨大消费潜力，增强消费对经济增长的拉动作用。

　　(四)进一步加快城镇化进程

　　做大做强中心城市。围绕提高综合竞争力、带动市域经济社会发展、建设最佳人居环境的目标，按照“一河穿城、两体推进、三点互动、四区共荣”的格局，进一步加快中心城市建设。新区内的公务员住宅小区一期、文昌大道、中原大道、东一路、东四路建成投用，广电中心、报业大厦、体育场馆、垃圾处理厂、沙北污水处理厂、新区水厂和大庆路沙河桥至建设路改造等基础设施工程开工建设。发挥经济开发区的体制优势，将其打造成项目建设平台、高新技术集聚地和改革开放的窗口，为全市提供示范辐射效应。加快旧城改造尤其是“城中村”和背街小巷的改造步伐，完成改造硬化小街小巷100条路街任务。建成五一广场。改造市人民公园。建成10处市区绿地。完成沙颍河周口城区段二期工程改造任务，建成6.9公里沙颍河沿河景观带，使防洪标准提高到20年一遇。做好南水北调周口供水工程前期工作。发挥公路、铁路、水路三位一体的交通优势，逐步确立中心城市的区域交通枢纽地位。加快房地产业发展，实施市区绿化和供暖工程，努力改善市民的居住生活环境。

　　加快区域性中心城镇发展。积极推进周项淮经济隆起带城乡一体化试点，抓紧研究制订发展规划，分步实施，扎实推进。搞好县城和39个国家、省、市重点小城镇建设，尽快形成特色和规模。抓好扶沟、沈丘、西华、郸城、鹿邑、商水等6个县的城区供水工程，建成淮阳、鹿邑、郸城、沈丘、太康等5个县的污水处理厂，开工建设西华、扶沟县的污水处理厂和商水县的排污设施，推进项城市向中等城市发展，县城向高标准小城市发展。加强小城镇交通、通讯、供水、供气、供暖、供电和污水、垃圾处理等基础设施建设，完善小城镇功能，形成一批现代化小城镇。坚持产业兴市兴镇，因地制宜地发展支柱产业和特色产业。加快生产要素向城镇集中，引导农村劳动力向城镇有序流动。

　　加强城市管理。克服“重建轻管”思想，完善中心城市“两级政府、三级管理、四级网络”管理体制，努力探索运用市场经济管理城市的办法，巩固城市公用事业改革成果。以创卫生城、园林城和文明城镇活动为载体，加强城市环境保护和生态建设。全面整治占道经营、店外经营和流动经营，依法拆除各类违章建筑，彻底根治卫生死角，搞好市容市貌的美化，提升城市品位。

　　(五)继续深化各项改革

　　抓好国企改革。继续巩固完善莲花集团、周口棉纺织印染厂的改制，重点抓好周口发电有限公司、宋河酒厂、邦杰集团的改制工作，引进战略投资者，国有工业企业全部实现产权多元化。加快商业、物资、供销等商贸流通企业的改革。继续完善国有资产有效监管体制，确保国有资产保值增值。

　　推进财税金融体制改革。不断深化部门预算、国库集中收付、政府采购、收支两条线和乡财县管、村财乡理改革，进一步优化支出结构，在保工资、保运转、保稳定的基础上，严格控制一般支出，集中财力办大事、办实事、办人民群众最关心的事，不断提高财政支出效益。推进商业银行、邮政储蓄机构及其他金融机构改革，尽快将城市信用社改制为商业银行。促进保险业规范有序发展，大力开发面向“三农”的保险产品。

　　大力发展非公有制经济。坚持“放心、放手、放开”的方针，把非公有制经济作为经济发展的主要增长点，认真落实促进非公有制经济发展的政策措施，营造非公有制经济发展的良好环境。建立健全以政府为主导、社会广泛参与的融资担保、创业辅导、法律政策服务等中小企业社会化服务体系，推动全市的中小企业和非公有制经济优化升级，加快发展。

　　加快文化体制改革。积极推进政企分开、政事分开和管办分离，加快国有文化企业公司制改造和经营性文化事业单位转企改制，发挥我市杂技、戏曲等民间文艺优势，鼓励发展民营文艺表演团体。

　　同时，继续深化国有粮食购销企业、住房制度、人事制度等改革，推进资源性产品价格和事业单位产权制度改革，进一步加快交通、城建、水利等领域的改革，明晰国有资产产权归属，努力向改革要效益，通过改革促发展。

　　(六)全面发展各项社会事业

　　加快科教文卫体等社会事业发展。大力发展高新技术产业。加快科学技术普及工作，推动企业技术创新。加快中药产业化基地建设，加大知识产权保护力度。巩固提高“普九”成果，全面实施素质教育。对农村义务教育阶段贫困家庭学生继续实施“两免一补”，提高农村中小学公用经费标准，改造农村中小学D级危房12万平方米，加快实施农村中小学远程教育工程，切实改善农村办学条件。优化整合教育资源，加强城市中小学和普通高中建设。逐步解决上学难、上学贵问题。大力发展成人教育、民办教育、职业教育和高等教育，完成周口职业技术学院扩建一期工程。大力整顿、规范药品价格和医疗服务秩序，强化医院管理。积极发展社区卫生服务事业，改造乡镇卫生院32所，努力缓解群众看病难、看病贵问题。继续搞好新型农村合作医疗试点工作，每人每年财政补贴标准由20元提高到40元。进一步完善公共卫生和医疗服务体系，搞好重大疫病预防控制。推动文化、体育、广播电视事业发展。推进统计改革和建设，提高统计数据的准确性和权威性，做好第二次全国农业普查工作。全面贯彻党的民族宗教政策，做好民族宗教工作。积极发展残疾人事业。重视做好老龄工作。继续做好物价、气象、地震、人防、消防、外事、侨务、史志等工作，促进社会事业全面进步。

　　坚持走可持续发展道路。继续实施农村部分计划生育家庭奖励扶助政策，强化对流动人口、下岗职工和无业人群的计划生育管理，稳定低生育水平，优化出生人口结构，提高出生人口素质，有效治理出生人口性别比偏高的问题。实行最严格的耕地保护制度，深入开展土地“三项整治”，合理开发、节约使用和有效保护水、土地等资源。强化环境和生态保护执法检查，重点抓好水污染防治、大气污染防治，坚决关停不能稳定达标排放的重污染企业，综合防治农业面源污染和畜禽养殖污染。加强城市环境综合整治，认真解决噪声扰民和垃圾围城现象。

　　(七)切实解决攸关民生的问题

　　实施积极的就业政策。多渠道开发就业岗位，支持发展社区就业和灵活就业。进一步完善和落实小额贷款、税费减免、就业培训等再就业优惠政策，建立健全公共就业服务体系和再就业援助制度。继续做好关闭破产企业职工、高校毕业生、新成长劳动力、被征地农民的就业指导和服务工作。确保新增城镇就业5万人。实现企业下岗职工再就业2.8万人，其中“4050”人员8000人。

　　进一步完善社会保障体系。扩大城镇社会保险覆盖面，加大保费征缴力度。完善城镇职工基本养老保险制度，改革基本养老金计发办法，确保企业离退休人员基本养老金按时足额发放。加强城镇基本医疗保险和失业、工伤、生育保险制度建设。提高城市居民最低生活保障标准，实现动态管理下的应保尽保。认真解决进城务工人员和失地农民的社会保障等问题。积极组织实施农村最低生活保障制度，将农村特困人口每人每月生活最低补助标准提高到20元。增加农村五保供养经费，“五保户”集中供养率达到20%以上。落实军烈属优抚政策。全面实施城乡医疗救助制度。积极发展社会福利事业和慈善事业，开展多种形式的捐助和帮扶活动。建立和完善地方粮食储备，保证宏观调控需要。

　　稳步提高居民收入水平和生活质量。提高城镇中低收入者的收入水平，严格执行最低工资制度。改革公务员工资制度，规范津贴、补贴。加大整村推进扶贫开发力度，解决10万农村贫困人口的脱贫和温饱问题。建成10个县市区城区利用天然气工程，着手解决市区供暖问题。开通周口至广州的长途客运列车，方便我市广大人民群众出行。认真清理建设领域拖欠工程款和农民工工资，努力实现清欠目标。进一步整顿和规范市场经济秩序，严厉打击制售假冒伪劣产品、商业欺诈等违法犯罪活动。加强食品药品安全监管。

　　做好安全生产和社会稳定工作。加强危险化学品、交通运输、烟花爆竹、特种设备及消防安全等领域和人员密集场所的专项安全整治，全面落实安全生产责任制，坚决遏制重特大事故发生。认真做好人民群众来信来访工作，充分发挥矛盾纠纷调处组织的作用，正确处理新时期社会矛盾。积极预防和妥善处置群体性事件。广泛深入开展平安周口建设，强化社会治安综合治理，完善社会治安防控体系，严厉打击各类刑事犯罪和经济犯罪，严密防范和严厉打击境内外敌对势力、邪教组织和各种暴力恐怖活动，维护国家安全，确保社会稳定。

　　(八)加强精神文明和民主法制建设

　　加强精神文明建设。深入开展“道德规范进万家、诚实守信万人行”活动。切实加强理想信念教育和思想道德建设，大力弘扬爱国主义、集体主义精神，继续宣传陈新庄、洪战辉等先进人物的典型事迹。实施公民道德建设工程，倡导公民基本道德规范。以伦理、心理、生理教育为载体，加强和改进未成年人思想道德建设以及大学生思想政治教育工作。繁荣发展新闻出版、广播影视、文化艺术和哲学社会科学。扎实开展“双百”评选、农村文明家庭、文明村组、文明村镇创建和信用村、信用户评定活动，组织好文化、科技、卫生“三下乡”活动。积极开展文明城市、文明景区、文明社区创建工作，继续开展科教、文体、法律、卫生“四进社区”活动。

　　坚持依法治市。继续开展普法教育，提高全民法律素质。自觉接受人大及其常委会的法律监督、工作监督和政协的民主监督，严格执行人大及其常委会的决议、决定，支持人大代表依法行使职权，认真办理人大代表建议和政协委员提案。广泛听取各民主党派、工商联和无党派人士的意见和建议，充分发挥工会、共青团、妇联等人民团体的桥梁纽带作用。加强法律援助，依法保障公民特别是妇女、未成年人和残疾人的合法权益。

　　积极支持部队和国防建设。加强国防教育，提高全民国防意识。满腔热情地关心、支持驻周解放军、武警部队、人民防空和民兵预备役建设，做好军队转业干部、复员退伍军人和随军家属安置工作。深入开展拥军优属和军民共建活动，进一步密切军政军民关系，争创全国双拥模范城。

　　四、加强政府自身改革和建设

　　近年来，我们不断推进服务型政府建设，行政能力和服务水平有了明显提高。但是还有一些地方和部门开拓创新不够，服务意识不强，工作效率不高，官僚主义、形式主义依然存在。我们必须适应新时期经济社会发展需要，进一步加强政府自身改革和建设，为人民服务、对人民负责、受人民监督、让人民满意。

　　加快政府职能转变。不断深化行政管理体制改革，继续推进政企分开、政资分开、政事分开，减少和规范行政审批。改善政府经济调节和市场监管职能，改变主要用行政手段管理经济的方式方法，着力为市场主体服务，创造良好发展环境。强化政府社会管理和公共服务职能，协调各种利益关系，建立健全预警和应急机制，提高保障公共安全和处置突发事件的能力。

　　全面推进依法行政。认真贯彻行政许可法和国务院依法行政实施纲要，严格依照法律规定的权限和程序行使权力、履行职责。坚持权力与责任挂钩、权力与利益脱钩的原则，约束行政权力，做到有权必有责、用权受监督、侵权要赔偿、违法要追究。搞好行政复议。加强行政执法监督，坚决纠正执法过程中的违法违纪行为。健全对重大事项决策的专家论证、技术咨询、决策评估制度，健全对涉及群众切身利益相关事项的决策公示、听证制度，推进决策科学化、民主化。严格执行问责制，提高政府执行力和公信力。充分发挥审计监督作用,强化对重点领域、重点部门、重点资金的审计监督,全面推行领导干部经济责任审计制度。加强行政效能监察。落实廉政建设责任制，深入开展反腐败斗争。今年要集中开展治理商业贿赂专项工作，重点治理工程建设、土地出让、产权交易、医药购销和政府采购等领域的商业贿赂问题，坚决纠正不正当交易行为，依法查处商业贿赂案件。继续纠正损害群众利益的不正之风，着力解决教育乱收费、医疗高收费等突出问题。

　　切实改进工作作风。认真实施公务员法，落实公务员内部正常的交流、转任和外部进入凡进必考制度，加强公务员教育、管理和监督，建设高素质的公务员队伍。积极整合行政资源，有效降低行政成本，切实提高行政效率。大力推进政务公开，在公共服务部门和单位全面推行办事公开制度。积极推进电子政务建设，建立和完善政府门户网站。健全行政服务大厅功能，精简办事程序，切实履行服务承诺。大兴求真务实之风，下决心治理文山会海，大力减少会议次数，压缩会议规模，改进会风、文风，开短会，讲短话，发短文，最大限度减少应酬和不必要的事务性活动。拿出更多的时间开展调查研究，深入了解社情民意，多为群众办实事、做好事、解难事。强化责任意识，狠抓工作落实，反对敷衍塞责，反对推诿扯皮。始终保持奋发有为、只争朝夕的精神状态，为“十一五”发展开好头、起好步，拼搏攀登，赢得主动。

　　各位代表，经过近几年的努力，我们已有了一个好的基础、一个好的氛围、一个好的势头。我们坚信，有以胡锦涛同志为总书记的党中央和省委、市委的坚强领导，有全市勤劳智慧、热爱家乡、不甘人后的一千多万人民的共同奋斗，2006年我们一定能够创造出更加辉煌的业绩，“十一五”宏伟蓝图一定能够如期实现，全市人民生活一定会更加幸福美好！
